# Is Obesity Policy in England Fit for Purpose? Analysis of Government Strategies and Policies, 1992–2020

**DOI:** 10.1111/1468-0009.12498

**Published:** 2021-01-19

**Authors:** DOLLY R.Z. THEIS, MARTIN WHITE

**Affiliations:** ^1^ Centre for Diet and Activity Research and MRC Epidemiology Unit University of Cambridge; ^2^ Bennett Institute for Public Policy University of Cambridge

**Keywords:** government, obesity, policy, policymaking, public health

## Abstract

**Policy Points**

- This analysis finds that government obesity policies in England have largely been proposed in a way that does not readily lead to implementation; that governments rarely commission evaluations of previous government strategies or learn from policy failures; that governments have tended to adopt less interventionist policy approaches; and that policies largely make high demands on individual agency, meaning they rely on individuals to make behavior changes rather than shaping external influences and are thus less likely to be effective or equitable.
- These findings may help explain why after 30 years of proposed government obesity policies, obesity prevalence and health inequities still have not been successfully reduced.
- If policymakers address the issues identified in this analysis, population obesity could be tackled more successfully, which has added urgency given the COVID‐19 pandemic.

**Context:**

In England, the majority of adults, and more than a quarter of children aged 2 to 15 years live with obesity or excess weight. From 1992 to 2020, even though the government published 14 obesity strategies in England, the prevalence of obesity has not been reduced. We aimed to determine whether such government strategies and policies have been fit for purpose regarding their strategic focus, nature, basis in theory and evidence, and implementation viability.

**Method:**

We undertook a mixed‐methods study, involving a document review and analysis of government strategies either wholly or partially dedicated to tackling obesity in England. We developed a theory‐based analytical framework, using content analysis and applied thematic analysis (ATA) to code all policies. Our interpretation drew on quantitative findings and thematic analysis.

**Findings:**

We identified and analyzed 14 government strategies published from 1992 to 2020 containing 689 wide‐ranging policies. Policies were largely proposed in a way that would be unlikely to lead to implementation; the majority were not interventionist and made high demands on individual agency, meaning that they relied on individuals to make behavior changes rather than shaping external influences, and are thus less likely to be effective or to reduce health inequalities.

**Conclusions:**

The government obesity strategies’ failure to reduce the prevalence of obesity in England for almost 30 years may be due to weaknesses in the policies’ design, leading to a lack of effectiveness, but they may also be due to failures of implementation and evaluation. These failures appear to have led to insufficient or no policy learning and governments proposing similar or identical policies repeatedly over many years. Governments should learn from their earlier policy failures. They should prioritize policies that make minimal demands on individuals and have the potential for population‐wide reach so as to maximize their potential for equitable impacts. Policies should be proposed in ways that readily lead to implementation and evaluation.

In england, the majority of men and women (67% and 60%, respectively) and more than a quarter of children aged 2 to 15 (28%) live with obesity or excess body weight.^1^ Living with obesity or excess weight is associated with long‐term physical, psychological, and social problems.^2^, ^3^ Related health problems, such as type‐2 diabetes, cardiovascular disease, and cancers, are estimated to cost England's National Health Service (NHS) at least £6.1 billion per year, and the overall cost of obesity to England's wider society is estimated to be £27 billion per year.^4^ The current COVID‐19 pandemic has brought to light additional risks for people living with obesity, such as an increased risk of testing positive for COVID‐19 and of hospitalization, as well as advanced levels of treatments and death.^5^

## Obesity Policy in the UK

**Figure 1:**
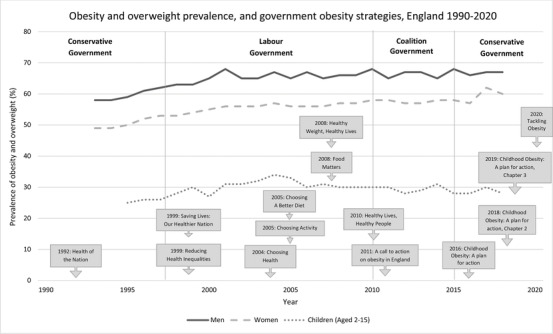
Timeline of Government Obesity Strategies and Prevalence of Obesity and Overweight in England (using Health Survey for England data)^1^

Obesity was first recognized by the UK government as a population health challenge in 1991.^6^ Since then, 14 government strategies (Figure 1), either wholly or partially dedicated to tackling obesity in England, have been published by four different governments. However, the prevalence of, and inequalities in obesity have not been successfully reduced, and the government faces ongoing criticism for failing to introduce effective policies.^7^, ^8^, ^9^

In 1998, the Labour government (1997‐2010) devolved power to the national assemblies in Scotland, Wales, and Northern Ireland, which included responsibility for formulating and implementing all health policies.^10^ Despite concordats aligning policies related to the National Health Service (NHS), population health, and other, wider health issues between the four national administrations, they are not legally binding, and each continues to measure and address health issues, including obesity, independently.^10^

The UK government defines government policy as “a course or general plan of action to be adopted by government, party, person etc.” and “statements of the government's position, intent or action.”^11^ Obesity policy is a particular type of health policy that “aims to impact positively on population health.”^12^ There are two main strands in health policy—health care and public health. The former is concerned with health care systems and the treatment of individuals, and the latter is concerned with the promotion of population health, the prolongation of life, and the prevention of ill health “through the organized efforts of society.”^13^ Public health policy recognizes that health outcomes are determined by more than an individual's behavior (e.g., by social and economic factors) and supports the integration of health across all sectors and policy areas.^12^, ^14^, ^15^, ^16^, ^17^ In England, this means that whereas the Department of Health and Social Care is primarily responsible for coordinating national obesity policy in England, particular policies can fall under the jurisdiction of other departments, such as the Department for Education or the Department for Transport.

### Obesity Policy Research

There is a growing body of research on obesity policy and related public health strategies.^6^, ^10^, ^18^, ^19^, ^20^, ^21^, ^22^, ^23^, ^24^, ^25^, ^26^, ^27^ Studies have examined whether policies are proposed in a way that “readily leads to implementation;”^6^, ^20^ the typology and framing of policies;^22^, ^23^, ^24^, ^25^, ^26^ issues related to regulation;^24^, ^28^, ^29^, ^30^ and the use of evaluation and evidence.^18^, ^19^, ^21^ To our knowledge, though, no comprehensive or systematic analysis of obesity policies proposed by government for England has been published. Instead, this research has been confined to a smaller selection of government‐proposed policies, to nongovernmental policy proposals, and/or to a particular analytical concept. The most directly relevant examination of obesity policy to date, by Jebb and colleagues (2013), provides an overview of obesity policy in England and the available evaluations. But it now is dated, does not include all proposed government policies and statements, and was a largely descriptive analysis.^6^

Given that the rates of obesity and overweight remain high in England with little sign of declining, a comprehensive analysis of government policies to date may provide valuable insights into the strategic approaches taken and their successes and failures, as well as to identify the potential for more effective obesity policies. We aimed to fill this gap in knowledge by answering two questions: What is the nature of the strategies and policies to tackle obesity in England that the governments have proposed to date? Were the strategies and policies fit for purpose in terms of their strategic focus, the policy measures included, their basis in theory and evidence, and their plans for implementation?

## Methods

We adopted a mixed‐methods approach using content analysis and applied thematic analysis (ATA) to investigate the governments’ strategy documents^31^, ^32^ before applying both qualitative and quantitative methods to the resultant data set, as explained next.

### Data Set and Acquisition

We undertook an analysis of government strategies either wholly or partially dedicated to tackling obesity in England. The term *strategy* refers to published government documents detailing an overall plan of action designed to achieve a long‐term aim, and the term *policy* refers to the individual principles, programs, and statements of intent or action contained in the strategies.^11^ We defined the data set as the distinct obesity policies nested in strategies wholly or partially dedicated to tackling obesity in England. Our time frame was set from when the UK government first formally recognized that it should introduce specific actions related to obesity, as published by the UK government (not devolved administrations) and containing policies that the government sought to introduce and/or had recommended, as well as policies that other sectors were expected to introduce and were readily accessible. For strategies partially focused on obesity, we included only those policies explicitly proposed as solutions to obesity and overweight. We included strategies and policies regardless of political party or the government department from which they originated or the sector at which they were targeted.

We identified government obesity strategies and individual policies contained in the strategies by searching the GOV.UK website, including relevant government department websites, and recorded them in a spreadsheet. We nested policies in their parent strategy and recorded and numbered them in the order in which they appeared. We recorded the year the strategy was published, the political party in government, the obesity reduction target (if any), and the individual policies (verbatim).

### Analytical Framework

To give our analysis a clear and comprehensive structure, we developed a theory‐based analytical framework using an iterative approach that involved multiple readings and codings of the data. Some themes were determined a priori to answer the prespecified research questions, drawing on published frameworks; others emerged during the analysis. Content analysis involves assigning codes or analytic categories to the data set, whereas ATA moves beyond description to interpretation by identifying, extracting, and interpreting “patterns of meaning in the data.”^31^, ^32^ Applied thematic analysis helps increase rigor and transparency in qualitative research—thereby decreasing the potential for impressionistic and biased results—and can flexibly accommodate the use of single, multiple, or no theoretical frameworks.^31^

We used frameworks to analyze strategies and policies that were identified in the existing literature or created new ones, which we included in the overarching analytical framework. For policies that did not fit into a framework's predetermined codes, we created new codes.^22^ After we had created a comprehensive coding system for the multiple themes, we refined it and checked it once more against the existing theoretical literature before drawing up a final coding map (for all themes, codes, and descriptions, see the Online Appendix). The main themes making up the framework are *target behavior type*, *policy type*, *implementation viability*, *regulation approach*, and *intervention agency demands*. Both authors agreed on the coding map.

For the *target behavior type*, we coded policies according to the broad behavior they sought to target (e.g., diet, physical activity, nonspecific). For *policy type*, we used the widely recognized Nuffield Foundation “Intervention Ladder” to characterize policies according to the extent to which they enabled or restricted choice.^6^, ^33^ We created new codes for policies that could not be characterized by the extent to which they enabled or restricted choice. These included *institutional*, *evaluation*, *research*, *guidance and standards*, and *professional development* policies (definitions in the Online Appendix). We separated *fiscal* and *non‐fiscal incentives* and *disincentives* into discrete codes to distinguish between taxation measures and other forms of incentives or disincentives such as a recognition award.^34^ The *do nothing or simply monitor the current situation* category became *monitor*, as we were unable to identify inaction in the strategies.

For the *implementation viability* theme, we identified the recurrent core components of existing frameworks that were used to assess the extent to which policies were conducive to implementation, including the World Health Organization's (WHO's) international framework.^20^, ^35^, ^36^ The core components of these frameworks applicable in this context were the specificity of the target population, the responsible actor, the presence of a monitoring and/or evaluation plan, the policy time frame, a statement of cost estimation and/or directly allocated budget for the policy, evidence cited to support the policy, and the identification of a theory of change to underpin the policy.^20^, ^35^

Different *regulation approaches* have been examined in obesity policy research, including self‐regulation by the food and drink industry, barriers to government regulation, and laws introduced to prevent obesity.^24^, ^28^, ^29^, ^30^, ^37^, ^38^ Regulation is not always law, however; it can also be an “act or process of controlling by rule or restriction.”^39^

To explore *regulation approach*, we analyzed the policies using Braithwaite's “responsive regulation pyramid.”^40^, ^41^, ^42^ Braithwaite helped shift the debate about business regulation away from a dichotomous dispute between deterrence‐based regulation and the removal of as many rules as possible, to one focusing on how regulators could achieve greater compliance and enforcement by understanding the context and motivation of those whose conduct they sought to regulate.^42^ The pyramid represents a four‐level regulatory approach, starting with *capacity building* at the base, in which regulatory actors learn about a problem and build their capacities to tackle it, then escalating to a *restorative approach* involving largely self‐regulation measures to “repair the harm that has been caused” by the problem.^42^ If not enough is done through self‐regulation, the strategy escalates to become more interventionist, and *deterrence* measures are introduced by the government or a regulatory body. Finally, in more extreme cases of inaction or insufficient action, *incapacitation* measures are introduced, for example, rescinding a license to operate. We assessed the policies for their stage of *regulatory approach* and whether regulatory escalation was proposed as part of the policy—for example, if government states that it will start with a self‐regulation approach and move to deterrence if self‐regulation is deemed ineffective.

Finally, to analyze the policies, we used the concept of *intervention agency demands*, which proposes that public health interventions differ according to the demands they make on an individual's *agency* (i.e., personal resources such as knowledge, engagement, and ability or power to act).^43^, ^44^ Since those interventions that make fewer demands on individual agency are likely to be the most effective and equitable, we used this analysis to find those policies most likely to reduce health inequity.^43^, ^44^

Backholer and colleagues offer a framework to assess the degree of agency required for an intervention to influence behavior change and the socioeconomic implications.^44^ As far as we know, this has not been used to systematically and rigorously examine government obesity policies at scale and over time. But in the absence of any other framework, we coded policies according to the framework's categorization and accepting that ongoing work may be required to refine and validate such a framework in the future.^45^ In relation to policies analyzed, we assigned a code only on the basis of the demands on the members of the population to whom a policy was directed, accepting that some policy interventions may also make demands on policymakers and professionals to ensure their implementation. We excluded from the coding those policy types with no clear or direct demands on individual agency (e.g., the appointment of a new minister).

We coded policy interventions that simply inform individuals about an issue and leave them to determine their preventive actions as being *agentic* and further coded them according to environmental level, that is, micro (schools, worksites, clinic, home) and macro (national, state, community). This category was represented by policies (e.g., information leaflets, social marketing campaigns) that require a high level of individual agency because people must notice (e.g., an informational leaflet), understand the information (usually requiring literacy, numeracy, or both), be motivated to change their behavior in response (e.g., choose healthier products), and then have the means and ability to do so.

**Figure 2:**
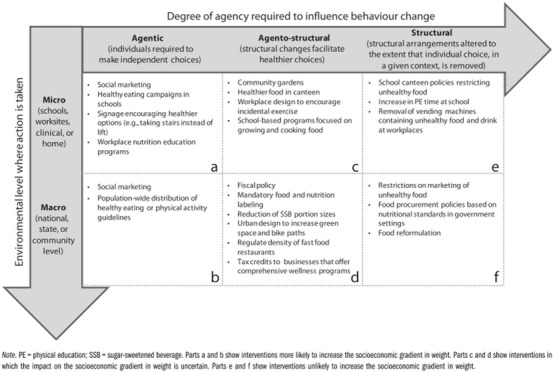
Backholer and Colleagues’ (2014) Framework for the Likely Impact of Obesity Prevention Strategies on Socioeconomic Inequalities in Population Weight^44^

At the other end of the spectrum are *structural* policies that eliminate or restrict choice and that therefore demand the least individual agency. Such policies include banning unhealthy food being advertised or sold somewhere, meaning that people are less exposed to unhealthy options and so are less likely to need to acquire and use resources to choose between these options. We also coded these according to environmental level. Finally, between the two ends of the spectrum are *agento‐structural* interventions, which account for the environment in which people behave and make choices but in which individual agency still plays an important role. An example is the provision of healthy food in a canteen or urban design to facilitate walking and cycling. We further coded these according to environmental level. Figure 2 presents Backholer and colleagues’ framework for the “likely impact of obesity prevention strategies on socioeconomic inequalities in population weight,” and the Online Appendix sets out the details of the six agency codes.^44^

### Data Analysis

In line with ATA, we first developed our analytical framework, as just described. Next, we analyzed all strategy documents to identify the specific policies they contained. Our analysis required multiple readings of the strategies to ensure that all individual policies had been identified according to the definition. We then undertook the initial thematic analysis and coding of the policies. Each policy was coded according to the categories of the five themes in our framework (see the Online Appendix) in an Excel spreadsheet. A single coder conducted the coding and thematic analysis. To check for interrater reliability, a second coder coded 10% of the policies, and both coders resolved any disagreements by discussion. Once the coding was complete, the number and proportion of codes were calculated for the policies overall, for each strategy, and for each government from 1992 to 2020. We then examined the results to identify patterns and meanings in the data, for example, changes over time and under different governments. We extracted examples from policy documents to illustrate our analysis.

## Results

We found 14 government strategies that fulfilled the inclusion criteria. Table 1 lists the strategies by month and year, political party in government, obesity reduction target(s) (if any), proportion of policies by *target behavior type* (diet, physical activity, or nonspecific), and the total number of policies. One strategy was published by the Conservative government (1979‐1997), seven by the Labour government (1997‐2010), two by the Conservative and Liberal Democrat coalition government (2010‐2015), and four by the Conservative government (2015‐2020).

**Table 1:** Government Obesity Strategies in England, 1992 to 2020

				**Policies by Target Behavior Type *n* (%)**			
**Political Party in Government**	**Month & Year**	**Government strategy**	**Obesity Reduction Target**	**Diet**	**Physical Activity**	**Nonspecific**	**Total Policies**
Conservative	July 1992	Health of the Nation^a^	By 2005, reduce proportion of obese men to 6% and obese women to 8%.	25 (58)	5 (12)	13 (30)	43
	Conservative Government Subtotal			25 (58)	5 (12)	13 (30)	43
Labour	July 1999	Saving Lives—Our Healthier Nation^a^	No target set.	7 (37)	4 (21)	8 (42)	19
	July 1999	Reducing Health Inequalities: an action report^a^	We recommend policies [for the] reduction of obesity.	10 (44)	6 (26)	7 (30)	23
	November 2004	Choosing Health^a^	Reduce obesity.	30 (28)	30 (28)	49 (44)	109
	March 2005	Choosing a Better Diet	By 2010, halt the year‐on‐year rise in obesity among children under 11.	53 (62)	0	33 (38)	86
	March 2005	Choosing Activity	By 2010, halt the year‐on‐year rise in obesity among children under 11.	0	67 (57)	51 (43)	118
	January 2008	Healthy Weight, Healthy Lives	By 2020, reverse the rising tide of obesity and overweight, and reduce the number of obese and overweight children to 2000 levels.	22 (31)	16 (22)	34 (47)	72
	July 2008	Food Matters^a^	Reduce the rate of increase in obesity among children under 11.	9 (100)	0	0	9
	Labour Government Subtotal			131 (30)	123 (28)	182 (42)	436
Coalition (Conservative and Liberal Democrat)	November 2010	Healthy Lives, Healthy People^a^	No target set.	6 (16)	11 (30)	20 (54)	37
	October 2011	A call to action on obesity in England	By 2020, achieve both a downward trend in the level of excess weight averaged across all adults, and a sustained downward trend in the level of excess weight in children.	13 (19)	13 (19)	43 (62)	69
	Coalition Government Subtotal			19 (18)	24 (23)	63 (59)	106
Conservative	August 2016	Childhood Obesity: a plan for action	Significantly reduce England's rate of childhood obesity within the next 10 years.	13 (43)	6 (20)	11 (37)	30
	June 2018	Childhood Obesity: a plan for action, Chapter 2	By 2030, halve childhood obesity rates and significantly reduce the health inequalities that persist.	24 (73)	3 (9)	6 (18)	33
	July 2019	Childhood Obesity: a plan for action, Chapter 3^a^	By 2030, reduce childhood obesity by 50%.	10 (42)	8 (33)	6 (25)	24
	July 2020	Tackling Obesity: government strategy	By 2030, halve childhood obesity rates.	9 (53)	0	8 (47)	17
	Conservative Government Subtotal			56 (54)	17 (16)	31 (30)	104
	Total			231 (33)	169 (25)	289 (42)	689

^a^ Public health strategies that include policies beyond obesity.

Seven of the strategies were broad public health strategies containing obesity as well as non obesity policies such as those for tobacco smoking and food safety. The other seven strategies contained only obesity policies, for example, diet and/or physical activity policies. Twelve of the 14 strategies contained obesity reduction targets, but only five of these were specific, numerical targets rather than statements like “aim to reduce obesity.” Strategies ranged from those containing 118 policies (*Choosing Activity*, 2005) to just nine (*Food Matters*, 2008). The median number of policies per strategy was 35.

Half of the strategies were set in the context of tackling health inequalities (*Saving Lives*, 1999; *Choosing Health*, 2004; *Choosing a Better Diet*, 2005; *Choosing Activity*, 2005; *Health Lives, Healthy People*, 2010; and *A call to action on obesity*, 2011). Although two (*Healthy Weight, Health Lives*, 2008, and *Food Matters*, 2008) discussed health inequality, the strategies’ purpose was not to tackle it. Three of the strategies (*Childhood Obesity: a plan for action, Chapter 2*, 2018 (*COP2*); *Childhood Obesity: a plan for action, Chapter 3*, 2019 (*COP3*); and *Tackling Obesity*, 2020) were meant to combat health inequality and included health inequality reduction targets. One strategy (*Reducing Health Inequalities*, 1999) concentrated on reducing health inequalities, and one (*Health of the Nation*, 1992) did not discuss health inequality or inequity at all.

Of those policies addressing health‐related behavior (diet or physical activity), a third were diet‐specific; a quarter were physical activity–specific; and the rest (42%) were nonspecific or included efforts to address both diet and activity, for example, the Healthy Schools Programme, which contains measures to improve both physical activity levels and diet in schools.

These strategies also contained a wide variety of *policy types*, from introducing food standards in schools and providing parents with support to live healthier lives to placing restrictions on television advertising of unhealthy products to children and reformulating unhealthy products (see Table 2). Many of the policies proposed were similar or exactly the same in multiple strategies over multiple years, often with no reference to having been proposed in a previous strategy. Only one strategy (*Saving Lives*, 1999) was based on a formal, independent evaluation of the previous government's strategy (*Health of the Nation*, 1992).^46^ Other strategies made more explicit references to previous strategies when there was a direct link between them. For example, *A call to action on obesity* (2011) references *Healthy Lives, Healthy People* (2010) because it proposes “building on the foundation laid down in the White Paper.”

**Table 2:** Number (%) of Policies by Policy Type in Government Obesity Strategies in England, 1992 to 2020

Government Strategy (Year)	Inst.	Eval.	Mon.	Research	Guidance or Standard	Profess Dev.	Elim. Choice	Restr. Choice	Fiscal Disincen.	Fiscal Incen.	Non‐fiscal Disincen.	Non‐fiscal Incent.	Change default	Enable	Inform	Total policies
Health of the Nation (1992)	6 (14)	2 (5)	2 (5)	4 (9)	8 (19)	4 (9)	0	0	0	0	0	0	2 (5)	7 (17)	8 (19)	**43**
Saving Lives—Our Healthier Nation (1999)	5 (26)	0	0	1 (5)	0	0	0	0	0	0	0	0	1 (5)	10 (53)	2 (11)	**19**
Reducing Health Inequalities: an action report (1999)	4 (17)	0	0	5 (22)	2 (9)	1 (4)	0	0	0	0	1 (4)	0	1 (4)	5 (22)	4 (17)	**23**
Choosing Health (1999)	13 (12)	7 (6)	1 (1)	4 (4)	24 (22)	14 (13)	0	4 (4)	0	0	1 (1)	3 (3)	3 (3)	17 (16)	18 (17)	**109**
Choosing a Better Diet (2005)	10 (12)	11 (13)	1 (1)	9 (10)	17 (20)	14 (16)	0	1 (1)	0	0	0	2 (2)	1 (1)	11 (13)	9 (10)	**86**
Choosing Activity (2005)	12 (10)	12 (10)	3 (3)	10 (8)	23 (19)	16 (14)	0	4 (3)	0	0	1 (1)	4 (3)	0	19 (16)	14 (12)	**118**
Healthy Weight, Healthy Lives (2008)	8 (11)	6 (8)	2 (3)	8 (11)	8 (11)	7 (10)	0	1 (1)	0	0	0	2 (3)	2 (3)	23 (32)	5 (7)	**72**
Food Matters (2008)	2 (22)	1 (11)	0	0	2 (22)	0	0	0	0	0	0	1 (11)	0	0	3 (33)	**9**
Healthy lives, Healthy people (2010)	6 (16)	1 (3)	1 (3)	2 (5)	4 (11)	3 (8)	0	0	0	0	0	2 (5)	0	15 (41)	3 (8)	**37**
A call to action on obesity in England (2011)	11 (16)	3 (4)	4 (6)	6 (9)	8 (12)	5 (7)	0	1 (1)	0	0	0	6 (9)	3 (4)	16 (23)	6 (9)	**69**
Childhood Obesity: a plan for action (2016)	1 (3)	2 (7)	0	4 (13)	6 (20)	6 (20)	0	0	1 (3)	0	0	2 (7)	2 (7)	6 (20)	0	**30**
Childhood Obesity: a plan for action, Chapter 2 (2018)	1 (3)	5 (15)	1 (3)	4 (12)	7 (21)	2 (6)	0	5 (15)	0	0	0	0	2 (6)	4 (12)	2 (6)	**33**
Childhood Obesity: a plan for action, Chapter 3 (2019)	1 (4)	3 (13)	1 (4)	6 (25)	2 (8)	0	0	0	1 (4)	0	0	0	2 (8)	5 (21)	3 (13)	**24**
Tackling Obesity: government strategy (2020)	4 (23)	0	0	2 (12)	0	1 (6)	0	2 (12)	0	0	0	0	2 (12)	2 (12)	4 (23)	**17**
Total	84 (12)	53 (8)	16 (2)	65 (9)	111 (16)	73 (11)	0	18 (3)	2 (0.3)	0	3 (0.4)	22 (3)	21 (3)	140 (20)	81 (12)	**689**

Abbreviations: Inst., Institutional; Eval., evaluation; Mon., Monitor; Profess Dev., Professional Development; Elim., Eliminate, Restr., Restrict; Disencen., Disincentive; Incen., Incentive.

Overall, the largest proportion of policies were *enable* policies (20%) such as the Healthy Start Programme, which provides vouchers for low‐income families to exchange for fresh fruit and vegetables and other products.^47^ There was also a relatively high proportion of *guidance or standards* policies (16%), aimed largely at the public sector, schools, and the NHS; *institutional* policies (12%), for example, the introduction of a ministerial position; *professional development* policies (11%), like training for health care professionals; and *inform* policies (12%), such as 5 A DAY.

In comparison, we found very few *fiscal* or *non‐fiscal disincentive* policies (0.3% and 0.4%, respectively); *monitor* policies such as weighing and measuring people regularly (2%); *restrict choice* policies like banning the promotion of unhealthy foods (3%); *change default* policies such as reformulation (3%); and *non‐fiscal incentive* policies like workplace awards for creating healthy environments (3%). No strategies proposed *fiscal incentives* (e.g., tax breaks on healthy products) or *eliminate choice* policies (e.g., banning an unhealthy product). Table 2 breaks down these *policy types* by government strategy and year.

The idea of *implementation viability* could be found in the majority of policies, with 71% suggesting a responsible agent, 57% setting a target population, 56% stating a theory of change, and 50% a time frame. But only 24% of the policies had any details of a monitoring or evaluation plan; only 19% cited any evidence to support the policy proposals; and only 9% offered any details about cost or included an allocated budget. We also looked at the proportion of all policies that fulfilled our seven implementation criteria and found that 197 policies—the largest proportion (29%)—did not fulfill a single one. This compares to only 59 policies (8%) that fulfilled all seven implementation viability criteria. For the rest of the policies, 75 (11%) fulfilled six criteria, 33 (5%) fulfilled five, 177 (25%) fulfilled four, 39 (6%) fulfilled three, 13 (2%) fulfilled two, and 96 policies (14%) fulfilled one.

Table 3 shows the distribution of *implementation viability* components by strategy. The Conservative government's strategies published between 2016 and 2019 contain the highest proportion of policies specifying a target population (87%, 94%, and 92%), and of these, *COP2* (2018) contains the highest proportion of policies with cited evidence (64%) and/or a theory of change (91%). Those strategies published by the Labour government between 2004 and 2005 contain the highest proportion of policies specifying a responsible agent (99%, 100%, and 99%) and a time frame (47%, 92%, and 93%). All these strategies contained a relatively low proportion of policies that specify a monitoring or evaluation plan (40% or less) and the estimated cost or a directly allocated budget (32% or less).

**Table 3:** Number (%) of Policy Proposals Identifying Implementation Viability Components in Government Obesity Strategies in England, 1992 to 2020

		**Number (%) of Implementation Viability Components**						
**Year**	**Government Strategy**	**Target Population**	**Responsible Agent**	**Monitoring or Evaluation**	**Time Frame**	**Cost/Budget**	**Evidence**	**Theory of Change**
1992	Health of the Nation	12 (28)	28 (65)	7 (16)	5 (12)	0	0	8 (19)
1999	Saving Lives—Our Healthier Nation	12 (63)	6 (32)	1 (5)	7 (37)	6 (32)	0	5 (26)
	Reducing Health Inequalities: an action report	15 (65)	4 (17)	3 (13)	6 (26)	4 (17)	0	0
2004	Choosing Health	69 (63)	108 (99)	43 (39)	51 (47)	2 (2)	18 (17)	80 (73)
2005	Choosing a Better Diet	51 (59)	86 (100)	35 (41)	79 (92)	17 (20)	38 (44)	58 (67)
2005	Choosing Activity	60 (51)	117 (99)	20 (17)	110 (93)	3 (3)	35 (30)	77 (65)
2008	Healthy Weight, Healthy Lives	41 (57)	15 (21)	16 (22)	19 (26)	13 (18)	8 (11)	44 (61)
	Food Matters	2 (22)	4 (44)	2 (22)	2 (22)	0	0	5 (56)
2010	Healthy Lives, Healthy People	21 (57)	31 (84)	5 (14)	8 (22)	7 (19)	1 (3)	14 (38)
2011	A call to action on obesity in England	26 (38)	44 (64)	8 (12)	18 (26)	2 (3)	0	28 (41)
2016	Childhood Obesity: a plan for action	26 (87)	19 (63)	9 (30)	9 (30)	4 (13)	3 (10)	12 (40)
2018	Childhood Obesity: plan for action, Chapter 2	31 (94)	24 (73)	9 (27)	19 (58)	1 (3)	21 (64)	30 (91)
2019	Childhood Obesity: a plan for action, Chapter 3	22 (92)	4 (17)	9 (38)	7 (29)	0	6 (25)	16 (67)
2020	Tackling Obesity: government strategy	8 (47)	2 (12)	0	4 (24)	0	4 (24)	6 (35)
Total		396 (57)	492 (71)	167 (24)	344 (50)	59 (9)	134 (19)	383 (56)

In our analysis of *regulation approach* (Table 4), a relatively high proportion of policies were capacity‐building policies with no indication of escalation (45%) or were restorative policies with no indication of escalation (39%). A much lower proportion of capacity‐building and restorative policies indicated regulatory escalation (8% and 3%, respectively). The proportion of deterrence policies with and without an indication of escalation was very low (1% and 4%, respectively), and we found no incapacitation policies. These findings show that the majority of government regulatory approaches in England (95%) have been capacity building and restorative, focusing on more voluntary measures that do not seek to deter actions.

**Table 4:** Number (%) of Policies by Regulation Approach in Government Obesity Strategies in England, 1992 to 2020

**Year**	**Government strategy**	**Capacity Building With Escalation**	**Capacity Building Without Escalation**	**Restoration With Escalation**	**Restoration Without Escalation**	**Deterrence With Escalation**	**Deterrence Without Escalation**	**Incapacitation**
1992	Health of the Nation	0	22 (51)	0	20 (47)	0	1 (2)	0
1999	Saving Lives—Our Healthier Nation	0	6 (32)	0	13 (68)	0	0	0
	Reducing Health Inequalities: an action report	0	11 (48)	1 (4)	11 (48)	0	0	0
2004	Choosing Health	4 (4)	47 (43)	3 (3)	47 (43)	3 (3)	5 (5)	0
2005	Choosing a Better Diet	21 (24)	37 (43)	3 (3)	22 (26)	1 (1)	2 (2)	0
2005	Choosing Activity	5 (4)	70 (59)	1 (1)	38 (32)	1 (1)	3 (3)	0
2008	Healthy Weight, Healthy Lives	2 (3)	35 (49)	3 (4)	31 (43)	0	1 (1)	0
	Food Matters	1 (11)	4 (44)	0	4 (44)	0	0	0
2010	Healthy Lives, Healthy People	2 (5)	14 (38)	1 (3)	20 (54)	0	0	0
2011	A call to action on obesity in England	4 (6)	29 (42)	0	32 (46)	0	4 (6)	0
2016	Childhood Obesity: a plan for action	2 (7)	14 (47)	1 (3)	10 (33)	0	3 (10)	0
2018	Childhood Obesity: a plan for action, Chapter 2	4 (12)	11 (33)	2 (6)	7 (21)	0	9 (27)	0
2019	Childhood Obesity: a plan for action, Chapter 3	5 (21)	8 (33)	1 (4)	9 (38)	1 (4%)	0	0
2020	Tackling Obesity: government strategy	3 (18)	3 (18)	2 (12)	5 (29)	1 (6%)	3 (18)	0
Total		53 (8)	311 (45)	18 (3)	269 (39)	7 (1%)	31 (4)	0

Until 2004, the governments’ regulatory policies had no indication of regulatory escalation, meaning that the policies were largely proposed without detailing what might happen in the case of insufficient action or change. Since 2004, more deterrence measures have been proposed, such as legislation on nutrition labeling for prepackaged foods, the Office of Communication Regulator's restriction of television advertising of unhealthy products, and a levy on sugary soft drinks. When the deterrence policies did indicate regulatory escalation, they were not suggesting introducing incapacitation measures but were indicating an extension of deterrence measures, like expanding the Soft Drinks Industry Levy to more products. *COP2* (2018) and the most recent *Tackling Obesity* (2020) had the highest proportion of deterrence policies (27% and 24%, respectively), and four strategies contained no deterrence policies (*Saving Lives*, 1999; *Reducing Health Inequalities*, 1999; *Food Matters*, 2008; and *Healthy Lives, Healthy People*, 2010).

We identified 312 policies that had the potential to affect *individual agency* but excluded the remaining 377 because they did not appear able to directly affect individual agency, such as the introduction of a ministerial position. Of the 312, we coded the largest proportion of policies (43%) as being agentic, meaning they would require individuals to draw on substantial personal resources to engage with an intervention effectively and would thus be less likely to be effective or equitable. Of these, 28% took place in a micro environmental level (e.g., school, worksite, clinic, home) and 72% at a macro level (e.g., national, local, community). The second largest proportion were agento‐structural (37%), and 19% were structural, meaning that they made the fewest demands on individual agency and thus were the most likely to be effective and equitable. A substantial majority (64%) of the structural interventions were voluntary, for example, the voluntary industry reformulation of unhealthy products, which research has shown tend to not meet intended objectives.^48^, ^49^ The voluntary nature of such interventions also highlights that agency with regard to interventions rests not only on the final target, as assessed by this scale (i.e., the population) but also on key stakeholders (e.g., commercial manufacturers and producers).

Table 5 shows the number (%) of policies in each strategy by the demands they make on individual agency, according to Backholer and colleagues’ framework for the “likely impact of obesity prevention strategies on socioeconomic inequalities in population weight.”^44^ The proportion of agentic and agento‐structural policies has remained relatively stable over the three decades. The proportion of structural policies was highest in the more recent *COP1* (2016), *COP2* (2018), and *Tackling Obesity* (2020) strategies (58%, 56%, and 40%, respectively), including banning the price and location promotions of unhealthy products and the introduction of a 9 pm watershed on unhealthy TV and online advertising.

**Table 5:** Number (%) of Policies by the Demands They Make on Individual Agency in Government Obesity Strategies in England, 1992 to 2020

		Agentic		Agento‐structural		Structural		
		Micro	Macro	Micro	Macro	Micro	Macro	
Year	Government Strategy	a	b	c	d	e	f	Number of Eligible Policies
1992	Health of the Nation	2 (9)	9 (41)	0	4 (18)	1 (5)	6 (27)	22
1999	Saving Lives—Our Healthier Nation	1 (8)	4 (31)	5 (38)	2 (15)	0	1 (8)	13
	Reducing Health Inequalities: an action report	1 (8)	5 (42)	2 (17)	1 (8)	2 (17)	1 (8)	12
2004	Choosing Health	7 (13)	15 (29)	12 (23)	8 (15)	6 (12)	4 (8)	52
2005	Choosing a Better Diet	2 (8)	13 (50)	5 (19)	2 (8)	1 (4)	3 (11)	26
2005	Choosing Activity	4 (10)	19 (45)	7 (16)	12 (29)	0	0	42
2008	Healthy Weight, Healthy Lives	7 (21)	5 (15)	11 (33)	8 (24)	0	2 (6)	33
	Food Matters	0	3 (60)	1 (20)	0	0	1 (20)	5
2010	Healthy Lives, Healthy People	7 (32)	6 (27)	4(19)	2 (9)	2 (9)	1 (4)	22
2011	A call to action on obesity in England	4 (12)	10 (29)	5 (15)	9 (26)	2 (6)	4 (12)	34
2016	Childhood Obesity: a plan for action	0	1 (7)	3 (21)	2 (14)	4 (29)	4 (29)	14
2018	Childhood Obesity: a plan for action, Chapter 2	0	1 (6)	0	6 (38)	1 (6)	8 (50)	16
2019	Childhood Obesity: a plan for action, Chapter 3	2 (18)	5 (46)	0	1 (9)	1 (9)	2 (18)	11
2020	Tackling Obesity: government strategy	0	1 (10)	2 (20)	3 (30)	0	4 (40)	10
Total		37 (12)	97 (31)	57 (18)	60 (19)	20 (6)	41 (13)	312

## Discussion

### Summary of the Main Findings

In this mixed‐method study, we identified all the government‐proposed obesity policies in England (*n* = 689) within obesity strategies (*n* = 14) over almost three decades (1992‐2020). We determined their nature and whether they had been adequate for their strategic focus, the policy measures included, their basis in theory and evidence, and their implementation plans. Using established theoretical frameworks and applied thematic analysis, we identified five main themes to define the nature of policies (*target behavior type, policy type, implementation viability, regulation approach*, and *intervention agency demands*).

A wide range of policy types were proposed, with a greater emphasis on diet than on physical activity. A substantial proportion of policies in all the strategies involved guidance or standards, professional development, institutional, informational, and enabling policies, indicating that governments have tended to prioritize the provision of information and capacity building in their obesity strategies rather than directly shaping the choices available to individuals through population‐level fiscal and regulatory measures, except for the more recent exceptions (e.g., the Soft Drinks Industry Levy).

Many of the proposed policies were similar or exactly the same in multiple strategies over multiple years, often with no reference to their presence in a previous strategy. Only one strategy (*Saving Lives*, 1999) commissioned a formal independent evaluation of the previous government's strategy (*Health of the Nation*, 1992).^46^ Few substantial changes in the proportions of the different *policy types* proposed appeared over time. The only non fiscal disincentives were proposed by the Labour government (1997‐2010). The more recent *COP2* (2018) and *Tackling Obesity* (2020) contain the highest proportions of restrictive policies (e.g., banning price promotions of unhealthy products), and *COP1* (2016) and *COP3* (2019) contain the only fiscal disincentive policies (e.g., the Soft Drinks Industry Levy).

Overall, these policies were not proposed in a way that could readily lead to effective implementation. The largest proportion of all policies (29%) did not fulfill one of the seven implementation viability criteria, compared to just 8% of policies that fulfilled all seven. Only 24% included a monitoring or evaluation plan; 19% cited any supporting scientific evidence; and only 9% included details about likely costs or an allocated budget. The majority of the policies contained a clear responsible agent (71%), a target population (57%), a theory of change (56%), and a time frame (50%).

In regard to regulation approach, a high proportion were capacity‐building policies (53%) and restorative policies (42%). The proportion of deterrence policies was very low (5%), and there were no incapacitation policies. Of the 312 policies that had the potential to make demands on individual agency, we assessed the largest proportion as being agentic (43%); that is, they require individuals to draw on substantial personal resources to engage effectively with an intervention and thus are unlikely to be effective and equitable. For the other eligible policies, 37% were agento‐structural and 19% were structural, meaning that they made the fewest demands on individual agency and were most likely to be effective and equitable. Given that 13 of the 14 strategies explicitly recognized the need to reduce health inequality, including one strategy that was primarily focused on reducing inequality in health and three that contained inequality reduction targets, the fact that only 19% of the policies proposed were likely to be effective in reducing inequalities is of great concern and accordingly may explain why efforts to reduce healthy inequalities have also widely failed.^50^, ^51^, ^52^ Furthermore, a substantial majority (64%) of the structural interventions were voluntary, for example, the voluntary industry reformulation of unhealthy products, which research has shown tend to not meet set objectives and so are even less likely to be effective or equitable.^48^, ^49^

### Strengths and Limitations

#### Strengths

Our analysis is the most comprehensive to date of government policies on obesity internationally, as it critically assesses all policies proposed by successive governments (*n* = 689) and explores how the nature of policies changed over an extensive period (28 years). We rigorously applied a theory‐based analytical framework and used ATA, which helps reduce the likelihood of bias by prioritizing a clear and systematic approach to the research while always maintaining a high level of transparency and reflexivity. Our method is also readily replicable, thus offering an opportunity for comparability with future research in the UK or elsewhere. The mixed‐methods approach enabled us to identify and present quantifiable patterns and for these patterns to be understood and interpreted through examples.

Our study updates previous analyses of obesity strategies in England^6^, ^21^ and offers a deeper and richer analysis, as it uses several different theoretical concepts, including the Nuffield Intervention Ladder, implementation frameworks, and Braithwaite's responsive regulation pyramid.^20^, ^22^, ^35^, ^53^ Our analysis can be compared to Haynes and colleagues’ study (2017), which examined stakeholder policy recommendations by their “impact on individual autonomy,” that is, how much individual liberty they were perceived to take away.^23^ Conversely, our study analyzed policies according to Backholer and colleagues’ framework assessing the degree of agency required for an obesity intervention to influence behavior change, which arguably presents a more positive way of perceiving the impact that policies have on individuals.^44^ For example, a policy deemed to remove individual liberty in Haynes and colleagues’ categorization is viewed as a policy that is empowering by removing the need for individuals to use their own resources in order to gain a healthy benefit. In regard to regulatory approach, we offered new empirical evidence of the regulatory approaches that UK governments have taken over almost three decades, a subject that has been the focus of much research.^24^, ^29^, ^37^, ^54^, ^55^

We presented a transparent coding scheme, which we encourage researchers to test and use to analyze other government strategies both in the UK and internationally. Only through the continual refinement and testing of coding frameworks like the one presented here can we reach a deeper, more comprehensive, and potentially generalizable understanding of government policy.

The use of various analytical themes demonstrates not only how these policies affect people at the individual level but also how they affect the responsible sectors. By analyzing the policies accordingly, we have demonstrated the need for those conceiving, designing, implementing, and evaluating policies to consider carefully both the intended and the unintended consequences and the implications of government policies on individuals and the responsible sector(s). For example, a policy designed to facilitate individual choice may require government to limit the choices of a responsible sector (e.g., mandatory menu labeling facilitates individuals’ choice by providing nutritional information, but at the same time it takes away the out‐of‐home food sector's choice of whether to implement it. This is justified on the grounds that it will have a net benefit for population health without major negative impacts on the commercial sector.^56^ Nonetheless, policymakers should seek to reconcile these implications during the design stage so as to minimize any potential negative unintended consequences.

As far as we are aware, ours is the first systematic and comprehensive analysis of government obesity policies according to an assessment of intervention demands on individual agency, hypothesized to be important for policy effectiveness and equity.^43^ Our categorization of policies according to agency was pragmatic, intuitive, and based on theory, but more work will be needed to refine and validate this framework.^45^ Although further refinement is strongly encouraged, we would argue that the assessment undertaken here has face validity and demonstrates the extent to which UK policies for obesity tend to be highly “agentic,” further signaling concern about their potential for both effectiveness and equitable impacts, even though 13 of the 14 strategies explicitly recognize the need to reduce health inequalities.^43^, ^44^

This study presents novel insights into the policies proposed within government obesity strategies in England, how they were proposed, and the implications for their implementation. For example, ours is the first study to find that the largest proportion of government obesity policies do not fulfill a single implementation viability criterion, with government strategies rarely suggesting obesity policies that formally cite scientific evidence or include a cost and/or budget or a monitoring or evaluation plan. It is the first to point out that only one UK government strategy formally evaluated a previous strategy, thereby revealing the lack of obvious learning from and evaluation of previous government actions. These findings have important implications for policymakers and may help explain why obesity levels have not been successfully reduced in England, despite the hundreds of government policies published over three decades.

#### Limitations

Our study analyzed policies only in government obesity strategies, which are only one type of policy document and one part of the policy process. We also favored breadth over depth, assessing 689 policies using a range of analytical lenses. We did not, however, conduct a deeper analysis of specific aspects of the policies, such as the quality of scientific evidence cited in policy documents and whether or how well policies were implemented. Given that 14 government strategies have been introduced during the last 28 years and yet have not reduced obesity rates, a deeper analysis would shed more light on the success or failure of these attempts at policymaking to address this major public health challenge. We did not conduct systematic searches for additional documentary material related to the policies we identified. This information might have been published in other government policy documents that could shed further light on the policies we studied. For example, we found that potentially important information was often missing from policy documents, such as the details of an evaluation plan. This may be because there was no evaluation plan or because it was published separately (e.g., by a government research agency). This is another avenue for research.

Instead of a mixed‐methods approach, we might have adopted a different—for example, more qualitative—approach to investigate the data more closely and leading to more detailed interpretations. But we felt that ours was the right approach as a first stage to get a sense of the breadth of the policies and their overall nature.

The coding was not always straightforward, as the policies could often be interpreted in several different ways. This links to the question of who is responsible and required to act in order to succeed in changing behavior. For example, mandatory menu labeling requires industry to change its behavior, as well as individuals to choose different options as a result of having nutritional information. This means that menu labeling could be deemed a restrict policy because it restricts industry from choosing whether to have menu labeling; it could be deemed an incentive policy as it encourages industry to reformulate and/or to offer more healthful food and drink; or it could be deemed an information policy if it focuses on how it gives individuals information.^57^ Our study concentrated on the demands that policies make on individuals in relation to agency. But it could have accounted for the demands that policies make on many actors when applicable. This also raises the question of whether a more sophisticated categorization of obesity policies is necessary (e.g., one that takes into account the agency demands on the responsible sector and on individuals, since many policies that benefit the health of individuals are perceived to have negative consequences on industry in regard to cost and freedom).^29^, ^30^, ^43^ Although Michie and colleagues’ “behaviour change wheel” recognizes the distinction between the responsible actor and the individual, their model does not explain what each policy requires from both the responsible actor and individual in order for the policy to be effective.^58^

Some policy programs were continued more explicitly over longer periods of time and across different governments (e.g., National Child Measurement Programme), whereas others appeared to be similar policies but actually had been rebranded in new strategies by successive governments (e.g., reformulation policies). It was beyond the scope of our study to analyze this aspect, but increasing the understanding of the extent to which different political parties eschew or embrace the same public health policy ideas, and why, could add much to our current knowledge.

### Interpretation and Implications for Policy and Practice

This study offers evidence that to date the UK governments have largely favored a less interventionist approach to reducing obesity, regardless of political party. The regulation approaches of the vast majority of policies (95%) were capacity building or restorative; that is, they focused on building the responsible actors’ capacity to deliver, or they trusted the responsible actors to reduce population obesity levels, regardless of any possible conflicts, such as the food industry's profiting from more food purchases. Governments may have avoided a more deterrence‐based, interventionist approach for fear of being perceived as controlling (the so‐called *nanny‐state*) or because they lacked knowledge about which more interventionist measures were likely to be effective.^59^

Braithwaite emphasized that more government intervention (i.e., deterrence) does not necessarily achieve greater compliance and that high compliance can be achieved without the use of deterrence measures like taxation.^53^ This could mean that less interventionist approaches that were deemed to have failed did not fail because they were less interventionist but because they did not fulfill the necessary conditions to achieve high compliance. Such policies would require a “networked” relationship between regulator and regulated, third‐party involvement (e.g., a public interest group) to prevent “regulatory capture,” such as when an industry or sector uses regulation to benefit private interests, thereby ensuring that those regulated have the capacity to deliver the policy, that consequences are meaningful and loopholes minimized, and that the process is transparent.^29^, ^41^, ^53^, ^60^

Earlier research showed the influence of neoliberal ideology, which advocates broad notions of personal responsibility, individual choice, free markets, and antigovernment intervention as barriers to public health policy.^61^, ^62^ Cullerton and colleagues found that recommending interventionist policies like legislation to tackle public health issues “creates tension within nations with a liberal tradition” because it is seen as taking away individual choice and impinging on individual and market freedoms.^62^ To navigate this tension, the UK government has looked to behavioral economics and “nudge” theory for solutions to change people's behavior without compulsion and consequently founded the Behavioural Insights Team in 2010 to inform policy.^63^

For example, *Healthy Lives, Healthy People* (2010) states that “the Government's approach to improving health and wellbeing … is therefore based on the following actions, which reflect the Coalition's core values of freedom, fairness and responsibility,” and includes policies such as the *Public Health Responsibility Deal*, which allowed the food and drinks industry to choose whether they delivered certain policies like labeling menus.^33^ Evaluations of the *Public Health Responsibility Deal* have shown why it failed to meet its objectives, largely because it did not fulfill conditions for effective self‐regulation policies, including being informed by evidence and being targeted, measurable, attributable, feasible, and with a time frame, as well as being independently and rigorously evaluated and transparently reported.^48^, ^64^

The vilification of government responsibility is commonly represented by the “nanny state” metaphor, which associates government intervention with “a fussing, over‐bearing nanny who intrudes into the private lives of citizens and treats them as infants who cannot be trusted to make their own decisions.”^61^ Swinburn and colleagues argued that although “genuine progress lies beyond the impasse of these entrenched dichotomies,” the strength of industry opposition and government reluctance to regulate presents a major barrier.^65^ Even though our study offers evidence that the UK government still favors a less interventionist approach in England, we also observed that this seems to be changing. This may be because government is recognizing that the existing approaches have not been effective and/or that more interventionist approaches are increasingly acceptable to the public.^66^ *COP2* (2018) and the most recent *Tackling Obesity* (2020) contain the highest proportions of deterrence measures, which may indicate a greater acceptance by the government, regardless of party ideology, of deterrence measures. But the stronger government interventions proposed in *COP2* (2018), such as legislation to mandate menu labeling, were not implemented before they were then proposed again in *Tackling Obesity* (2020) only two years later. This demonstrates that policy proposals do not automatically lead to implementation, even within a two‐year period. More worrying is the observation that the same policies can be proposed with no reference to having been previously proposed but not implemented.

Our study has shown that policies are largely proposed in a way that would not readily lead to implementation and that only five strategies set a specific numerical obesity reduction target. This may help explain why such policies have not yet reduced obesity prevalence and health inequities.^20^, ^26^, ^35^ No matter how well‐intended and evidence‐informed a policy is, if it is nebulously written without a clear target, it makes implementation difficult, and it is unlikely the policy will be deemed successful.^35^ The lack of such basic information as the cost of certain policies was further highlighted in a recent National Audit Office report on the UK government's approach to tackling childhood obesity in England, which found that the Department of Health and Social Care did not know how much the central government had spent tackling childhood obesity.^67^ This raises a number of questions. For example, do governments deliberately propose policies in such a way, or is it a fault of the policy process? If the former, then perhaps proposing policies serves a more political purpose of being seen to be acting, that is, a rhetorical rather than a meaningful commitment. But if it is the latter, then what is the purpose of proposing policies at all if they are unlikely to lead to implementation?

The lack of government clarity and the information about the potential effectiveness, implementation, and cost of its own policies may be further compounded by an apparent aversion to conducting high‐quality, independent evaluations (which risk demonstrating failure, as well as success), which in turn may reduce a government's ability to learn lessons from past policies.^21^ Baggott found that England's public health policies either tightly controlled their evaluation in order to minimize criticism, were not conducted at all, or were conducted in a way that made lessons for future policymaking ambiguous.^21^

The time it takes to put together a strategy may also explain why policies are often proposed without information that would make them more likely to be implemented. For example, it was announced in May 2020 that Prime Minister Boris Johnson would be publishing a new government obesity strategy, which was then published two and a half months later.^68^, ^69^ This could be considered insufficient time to prepare a highly implementable government strategy. Nonetheless, the majority of policies proposed in 2020 had already been proposed in earlier strategies, such as *COP2* (2018), but were never implemented. Thus the government, as well as associated agencies like Public Health England, should have had sufficient time to prepare fully developed implementation plans. And if they had already developed implementation plans, then why were these not included in the strategy?

### Unanswered Questions and Future Research

Our study points to questions that are critical to tackling rising levels of obesity internationally, such as the question of what we *should* expect from a government obesity strategy. Furthermore, if numerous strategies have been introduced without reducing obesity, then what aspects of the approaches used to date are not working, and what should be prioritized instead?

From almost any point of view, it seems reasonable to expect that government policy should have sufficient scope to address the problem under consideration, that it should be based on the best available theory and evidence, and that it should be proposed and implemented in such a way that it can be effective. All these aspects of policymaking were found wanting in obesity policy in England. Foresight's report, *Tackling Obesities: Future Choices* (2007), contended that obesity is a systemic challenge and that to stand any chance of reversing current trends, effective interventions across a wide range of fronts would be necessary. To date, such policies have been limited in their focus (regarding the range of systemic drivers of obesity identified by Foresight) and have had far too great a focus on downstream interventions, with individual behavior change framed as a “choice” agenda and insufficient emphasis on upstream population interventions. Both theory and evidence to support population interventions have grown over the 13 years since the Foresight report (2007) and, in the UK at least, we are starting to see the beginnings of a move toward population‐level policies.^17^

Yet even the most recent policies have been criticized for addressing an insufficient number of fundamental drivers of obesity to be able to make a significant impact on the population and for representing relatively weak levers for change. Obesity arises as an unintended consequence of those food, transport, work, and leisure systems designed primarily for other purposes (most notably economic growth).^17^ The result is obesogenic environments, which encourage people to overeat and be physically inactive. Because these systems are complex and adaptive, when we intervene in them, they change to achieve a new equilibrium, a point at which prior goals can still be achieved. Thus if a fiscal policy reduces profits (and sector growth) from unhealthy foods, the food industry will rapidly mitigate the impact of the tax by finding new ways to increase profits and growth, which might negate or undermine the effects of the public health policy.^56^ This has been described in other fields as the “balloon effect.”^70^

**Figure 3:**
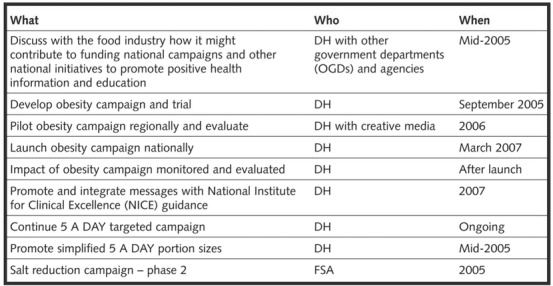
Implementation Table for Education Policies in Choosing a Better Diet (2005)^72^

Our analysis uncovered both good and poor policymaking from the perspective of enabling implementation. Figure 3 shows how the Labour government's policies were proposed in its *Choosing a Better Diet* strategy (2005). It explains clearly what the policy is, who is responsible, and when the policy will be implemented.^71^ Such an approach could be extended in future government strategies to include additional criteria that would better ensure that policies are more readily implementable, such as evaluation plans and costs. Further research could evaluate the impact of such a framework.

We did not analyze the quality of the evidence cited in the strategies, so future research could explore the quality and type of evidence cited and whether the proposed policies align with the highest‐quality evidence available at the time. Linked to this is how policies are framed based on evidence and understanding. We did not analyze the way policies were framed and on what arguments they were based. Future research could examine the framing of proposed policies by different policymakers and explore whether this is linked to the types of policies proposed and changes over time or under different governments.

It will be important to distinguish the failings of the policies themselves from implementation failures. The failure to reduce obesity rates in England, despite so many strategies being published, could be because of the partial or complete failure of the policies' implementation. Future research could explore the extent to which policies were implemented and in what ways they differed from those proposed but not implemented.^36^ Another explanation could be the regulatory approach taken. In this study, we have presented an important starting point in analyzing the implications of different regulatory approaches. A more in‐depth analysis of regulatory approaches taken by the government might help spell out the relationship between policy types, regulatory approach, compliance levels, and associated outcomes.

Future research should also explore who or what is behind the formulation of government obesity policies and strategies, so as to generate a greater understanding of the policy process itself. This may help explain why certain policies are favored over others, what barriers and facilitators appear in the policymaking process, why some people are more influential than others, and why policies are proposed with or without supporting evidence.

## Conclusions

Our study has provided new evidence that in almost 30 years, the UK government has proposed 689 wide‐ranging policies to tackle obesity in England but has not yet successfully and consistently reduced obesity prevalence or health inequities. Only one of the 14 government strategies commissioned an independent evaluation of previous government strategies for obesity, which suggests a significant deficit of government policy learning and may explain why similar or identical policies are put forward multiple times over many years.

Many of these policies were set out in a way that does not readily lead to implementation, and the largest proportion of policies did not fulfill any of the implementation viability criteria. Overall, governments have adopted less interventionist policy approaches, although this has changed in recent years. The policies have relied on a design that makes high demands on individual agency, meaning that they rely on individuals to make behavior changes rather than shaping external influences such as the environment or economy and are thus less likely to be effective or reduce health inequities. We found that a wide range of inadequacies related to government obesity policies are likely to explain why governments have repeatedly failed to reduce the inequalities in, and the prevalence of, obesity.

To increase the likelihood of policies being implemented, governments should accompany policy proposals with information ensuring they can readily lead to implementation, such as a clearly identified responsible agent, evaluation plan, and time frame; and to increase the likelihood of effectiveness and equitability, governments should increasingly focus obesity strategies on “low agency” population intervention policies that more comprehensively address the most powerful levers for system change.

*Funding/Support*: The research is funded by/supported by a PhD studentship awarded to Dolly R.Z. Theis by the National Institute for Health Research (NIHR), School for Public Health Research (Grant No. PD‐SPH‐2015‐10025). Martin White was supported by the MRC Epidemiology Unit (Grant No. MC_UU_00006/7) and the Centre for Diet & Activity Research (CEDAR), with funding from the British Heart Foundation, Cancer Research UK, Economic & Social Research Council, Medical Research Council, NIHR and Wellcome Trust (Grant Nos. ES/G007462/1, MR/K023187/1 & 087636/Z/08/Z). The views expressed are those of the authors and not necessarily those of any of the aforementioned funders.

*Acknowledgments*: We thank Kate Ellis for independently coding the data. We thank Dr Dennis Grube, lecturer in public policy in the Department of Politics and International Studies (POLIS), University of Cambridge, and Dr Kathryn Oliver, associate professor of sociology and public health at the London School of Hygiene and Tropical Medicine, for insightful comments on this article.

*Conflict of Interest Disclosures*: Both authors completed the ICMJE Form for Disclosure of Potential Conflicts of Interest. No conflicts were reported.

## Supporting information

Online Appendix
