# Supplementary material for: Is Obesity Policy in England Fit for Purpose? Analysis of Government Strategies and Policies, 1992–2020
Source: Milbank Q. 2021 Jan 19;99(1):126–70. doi: 10.1111/1468-0009.12498 (PMC7984668; doi:10.1111/1468-0009.12498)
Supplement: Supplementary file 1 — Online Appendix [file MILQ-99-126-s001.docx]

**Online Appendix**

**Coding Framework**

| **Theme** | **Code** | | **Description** | **Examples** |
| --- | --- | --- | --- | --- |
| **Target behavior type** | **Diet** | | Any policy focused solely on addressing diet. | Reformulation of unhealthy food and drink, menu labeling, provision of fruit and vegetables. |
|  | **Physical activity** | | Any policy focused solely on addressing physical activity. | Provision of cycle infrastructure, compulsory physical education. |
|  | **Nonspecific** | | Any policy that does not specify whether it seeks to tackle diet or physical activity and any policy focused on addressing both diet and activity. | Change4Life, Sure Start. |
| **Policy type: Code by immediate aim, that is, where policies have two or more types of aim, for example, produce research on an inform intervention, code according to the initial type, for example, code the aforementioned example as research rather than informing policy.** | **1. Institutional** | | Any policy relating to institutional change in national or local government or any other sector, body, or organization. Includes a policy to introduce a new or update an existing strategy. | A change in or a new ministerial position, government body, organization, or strategy (eg, in a specific policy area). |
|  | **2. Evaluate** | | A policy focused solely on an evaluation carried out by government, an independent body, or another nongovernment sector or organization. Includes the evaluation of a policy program or other initiative. | Evaluation of a particular policy, a government review. |
|  | **3. Monitor** | | A policy relating to the monitoring and/or surveillance of an identified issue, for example, population obesity levels. Includes any policy that seeks to continue and/or expand an existing monitoring/surveillance program. | National Health Survey for England, National Child Measurement Programme, Central Health Monitoring Unit. |
|  | **4. Research** | | Any policy focused on the facilitation, funding, or initiation of research on an identified issue by government or any other sector, body, or organization. Includes any policy to produce a one-off piece of research on an issue. | NIHR Obesity Policy Research Unit, launch of a national prevention research initiative. |
|  | **5. Guidance or standards** | | Any policy relating to the development, implementation, or updating of guidance or standards by and/or for government or any other sector, body, or organization. Standards are sometimes referred to as “codes” or “codes of conduct.” | NICE guidance, school food standards, government buying standards. |
|  | **6. Professional development** | | Any policy relating to the development or training of relevant professionals. | Health professionals, NCMP training, resource packs for teachers, training for planners on the health implication of local plans. |
|  | **7. Eliminate choice** | | A policy that seeks to regulate in such a way as to entirely eliminate a choice. | Ban a particular food or drink; ban transfats. |
|  | **8. Restrict choice** | | A policy that regulate to restrict options available to people (including to certain demographics). | Ban the sale of energy drinks to children; ban vending machines in schools; ban advertising of junk food to children on TV. |
|  | **9. Fiscal disincentive** | | Any policy that uses a fiscal disincentive to achieve change or reduce noncompliance. | Soft Drinks industry Levy. |
|  | **10. Fiscal incentive** | | Any policy that uses a fiscal incentive to achieve change or increase compliance. | Tax break on bicycle purchases for employees, tax cut for the production or sales of healthy products. |
|  | **11. Non-fiscal disincentive** | | Any policy that uses a non-fiscal disincentive to achieve change or reduce noncompliance. | Traffic congestion charge.^73^ |
|  | **12. Non-fiscal incentive** | | Any policy that uses a non-fiscal incentive such as a reward or award to achieve change or increase compliance. | Healthy Eating Award, Healthy Workplace Award. |
|  | **13. Change default** | | Any policy that seeks to change the default of a product by making it healthier or when options are still offered, the default option is the healthier of them. | Calorie and sugar reduction program. |
|  | **14. Enable** | | Any policy that enables individuals to change their diet and/or physical activity behavior. Differs from non-fiscal incentive policies in that the offering is passive. | Weight loss classes, free fruit and vegetables, Our Family Health digital support, Cooking for Kids. |
|  | **15. Inform** | | Any policy that seeks to provide people with information, including through a health promotion campaign. | Menu labeling, food labeling, health leaflet, 5 A DAY. |
| **Implementation viability** | **Target population** | | When the target population is specified enough to know who is included and who is not. | Children, women, low-income groups, families, ethnic groups, parents. |
|  | **Responsible agent** | | A policy in which the responsible agent is specified enough to know which individuals or organizations will be responsible. “Government” or “the food industry” was not considered specific enough. For example, there are many departments in government and many companies in the broader food industry, so the responsible one(s) needs to be made explicit. | Department of Health and Social Care, Sport England, Office for Standards in Education, Children’s services and Skills (Ofsted). |
|  | **Monitoring and/or evaluation** | | A policy in which details of a monitoring and/or evaluation plan are made explicit in relation to a policy. | Evaluate Healthy Start Scheme before rolling out; PHE will provide an assessment at 18 and 36 months on the approach . . . [and] use this information to determine whether sufficient progress is being made. |
|  | **Time frame** | | A policy in which details of a time frame, including when a start or implementation date is stated, are made explicit. | We will consult [on the SDIL] before the end of 2018 on our intention to introduce legislation; HM Treasury will . . . legislate in the finance bill 2017. |
|  | **Cost and/or budget** | | A policy in which details of the anticipated, estimated, or calculated cost and/or budget are made explicit. A budget was counted only when it was in direct reference to a policy. | Sport England will receive £392 million from the government and an estimated £324 million from lottery funding from 2008 to 2011 to deliver community sports.^47^ |
|  | **Cited evidence** | | When a policy proposal was supported by cited scientific evidence of any kind, either in a reference or directly referred to in the text of the policy proposal. | Evidence tells us that one of the reasons energy drinks are appealing to children is that they are often cheaper than soft drinks (with cited reference).^74^ |
|  | **Change theory** | | When a policy was proposed alongside a proposed or established theory relating to changes in the target group, that is, some form of explanation relating to how the policy will or is designed to achieve a desired outcome(s) and how the target group will do that. | Choosing A Better Diet sets out a theory relating to changes in individuals’ food choices: if people are provided with simpler and clearer labeling that is “more in keeping with their lifestyles,” then they will be able to make healthier food choices.^72^ |
| **Regulation approach** | **Capacity building** | | Any policy that builds the capacity and/or knowledge of an issue, including for the government, any other sector, organization, or body, or the public. | With funding from the regional directors of Public Health, they set up a working group with the entertainment technology industry to develop tools for parents to manage children’s time online.^47^ |
|  | **Restoration** | | Any proposed and/or recommended policy that is based on the assumption that the responsible actor(s) is able and/or willing to act without deterrence measures, that is, various forms of self-regulation by government, any other sector, organization, or body or individuals. The process may involve public praising or shaming for action or inaction. | Public Health Responsibility Deal,^75^ and calorie and sugar reduction program.^74^ |
|  | **Deterrence** | | Any policy that uses deterrence measures to prevent or control certain conduct, based on the assumption that the responsible actor(s) is unable and/or unwilling to act without deterrence. The deterrence tool may be the responsibility of government and/or any other sector, organization, or body. Consequences may be financial, legal, or otherwise. | Soft Drinks Industry Levy,^76^ and The Office of Communications (Ofcom) advertising restrictions.^77^ |
|  | **Incapacitation** | | Any policy that incapacitates government, any other sector, organization, or body, or individual from acting or operating in a certain way or at all. | Revoking a food company’s license to sell its products, and making the selling and/or consumption of a product illegal. |
| **Intervention agency demands** | **Agentic (micro)** | a | Any policy targeted at the micro level (eg, school, worksite, clinic, or home) that demands a high level of individual agency, meaning that it requires individuals to draw on high levels of personal resources (eg, knowledge, engagement, willingness) to engage with the intervention effectively. Includes any policy that requires individuals to actively engage rather than be proactively engaged with. | Healthy eating campaign in school, health leaflet in a clinical setting, and workplace nutrition education program. |
|  | **Agentic (macro)** | b | Any policy targeted at the macro level (eg, national, local, or community) that demands a high level of individual agency, meaning that it requires individuals to draw on high levels of personal resources (eg, knowledge, engagement, willingness) to engage with the intervention effectively. Includes any policy that requires individuals to actively engage rather than be proactively engaged with. | National social marketing campaign (eg, Change4Life), population-wide healthy eating or physical activity guidelines. |
|  | **Agento-structural (micro)** | c | Any policy targeted at the micro level (eg, school, worksite, clinic, or home) that makes low to moderate demands on individual agency, meaning that it requires individuals to draw on low to moderate amounts of personal resources (eg, knowledge, engagement, willingness) to engage with the intervention effectively. Includes any policy that offers proactive support to individuals to change their own behavior. | Community gardens, healthier food served in canteens, workplace design to encourage healthier behaviors, school-based cooking programs. |
|  | **Agento-structural (macro)** | d | Any policy targeted at the macro level (eg, national, local, community) that makes low to moderate demands on individual agency, meaning that it requires individuals to draw on low to moderate amounts of personal resources (eg, knowledge, engagement, willingness) to engage with the intervention effectively. Includes any policy that offers proactive support to individuals to change their own behavior. | Healthy voucher program (eg, Healthy Start Scheme), regulation of fast-food outlets, mandatory menu labeling, policy planning to increase walking and cycling infrastructure, reduction of portion sizes of, for example, sugar-sweetened beverages. |
|  | **Structural (micro)** | e | Any policy targeted at the micro level (eg, school, worksite, clinic, home) that makes no obvious or minimal demands on individual agency, meaning that it requires individuals to draw on no or minimal personal resources (eg. knowledge, material resources, engagement, willingness) to engage with the intervention effectively. Includes any policy that individuals may not notice that the decision or change has been made without any need for their engagement. | School or workplace canteen policies restricting unhealthy food, mandatory school food standards, mandatory increase of physical education and activity delivered in schools, and removal of vending machines containing unhealthy products from the workplace. |
|  | **Structural (macro)** | f | Any policy targeted at the macro level (eg, national, local, community) that makes no obvious or minimal demands on individual agency, meaning that it requires individuals to draw on no or minimal personal resources (eg, knowledge, material resources, engagement, willingness) to engage with the intervention effectively. Includes any policy that individuals may not notice that the decision or change has been made without any need for their engagement. | Banning the sale of energy drinks to children; regulating unhealthy food advertisements or marketing; food procurement policies based on nutritional standards (eg, government buying standards for food, food reformulation policies. |
